# Feasibility of Intraoperative Intraspinal Endosonography Using a Miniaturized Ultrasound Probe Through an Extended Interlaminar Lumbar Approach: A Pilot Study

**DOI:** 10.3390/jcm15114090

**Published:** 2026-05-25

**Authors:** Ralf Stroop, Samer Zawy Alsofy, Makoto Nakamura, Moritz Wegner, Christian Ewelt

**Affiliations:** 1 Faculty of Health, Department of Medicine, Witten-Herdecke University, 58455 Witten, Germany; szawyalsofy@barbaraklinik.de; 2 Department of Human Medicine, Faculty of Medicine, Medical School Hamburg, 20457 Hamburg, Germany; 3 Department of Neurosurgery, Academic Hospital of University Münster, St. Barbara-Hospital, 59073 Hamm, Germany; cewelt@barbaraklinik.de; 4 Department of Neurosurgery, Academic Hospital Köln-Merheim, Witten-Herdecke University, 51072 Köln, Germany; nakamuram@kliniken-koeln.de; 5 Department of Vascular and Endovascular Surgery, Faculty of Medicine, University Hospital Cologne, University of Cologne, 50937 Köln, Germany; moritz.wegner@outlook.de

**Keywords:** intraoperative ultrasound, spine surgery, lumbar decompression, microdiscectomy, minimally invasive surgery, endosonography, nerve root

## Abstract

**Background/Objectives:** Intraoperative ultrasound was explored in the 1980s to assess lumbar spinal decompression; however, conventional probes require large bony windows and are poorly suited for minimally invasive surgery. This technical note evaluates the feasibility of intraoperative intraspinal endosonography (IOISES) using a miniaturized linear ultrasound probe introduced directly into the spinal canal through a microsurgical access corridor. **Methods:** This observational feasibility study included two patients undergoing lumbar spine surgery (microdiscectomy for disc herniation and decompression for spinal stenosis). After decompression and hemostasis, a miniaturized linear probe (Fujifilm L51K) connected to an Arietta A65 system was inserted into the spinal canal via an extended interlaminar approach. Imaging was performed prior to wound closure. The primary outcome was the technical feasibility of probe insertion and image acquisition. The secondary outcomes included intraoperative usability, visualization of neural structures, and integration into the surgical workflow. **Results:** Probe insertion and imaging were successful in both cases (100%). IOISES enabled high-resolution visualization of the dural sac and nerve roots, allowing intraoperative visualization of the extent of decompression. Probe handling and rotation were feasible without forced manipulation. No adverse events occurred, and the technique was integrated into the surgical workflow without prolonging operative time. **Conclusions:** IOISES is technically feasible and enables real-time intraspinal visualization during minimally invasive spinal surgery. This approach represents a shift from extraspinal to intraspinal ultrasound imaging. Further studies are required to evaluate reproducibility and clinical impact.

## 1. Introduction

Intraoperative spinal ultrasound was first described by Gooding et al. in 1984 as a tool for assessing dural and neural decompression during lumbar laminectomy [1]. Subsequent studies confirmed its diagnostic utility for evaluating decompression and identifying residual compressive pathology [2,3]. However, conventional ultrasound probes are applied extraspinally and require sufficiently large bony windows to achieve adequate acoustic coupling and visualization. This limitation restricts the applicability of intraoperative ultrasound in modern minimally invasive spinal surgery, where surgical corridors are intentionally kept narrow to preserve spinal stability and reduce tissue trauma. The extended interlaminar lumbar approach, as used for microdiscectomy and microsurgical decompression, provides a keyhole corridor that is typically sufficient for direct neural decompression but does not accommodate conventional ultrasound probes for intraoperative imaging.

Already in the early description of intraoperative spinal ultrasound, Gooding et al. noted that the technique would require pencil-sized ultrasound probes to become compatible with microsurgical spinal approaches. Despite this early recognition, the gap between conventional probe dimensions and microsurgical access corridors has persisted as a major barrier to clinical adoption in minimally invasive spine surgery.

Recent advances in ultrasound probe miniaturization have enabled the development of high-frequency linear probes with substantially reduced dimensions. In parallel fields, miniaturized transducers have been successfully integrated into catheter-based systems such as intravascular ultrasound (IVUS) and endobronchial ultrasound (EBUS), demonstrating the feasibility of high-resolution imaging in confined anatomical spaces [4,5]. These developments suggest that similar miniaturization may allow direct intraspinal introduction of ultrasound probes through microsurgical interlaminar corridors, potentially overcoming the anatomical and technical limitations of traditional extraspinal ultrasound approaches.

To our knowledge, no prior study has described intraspinal ultrasound imaging using a miniaturized probe introduced through a microsurgical lumbar access corridor. The present study introduces the technique of intraoperative intraspinal endosonography (IOISES) and describes its technical implementation using a miniaturized linear probe. The aim of this study was to evaluate the initial technical feasibility of IOISES, including probe insertion, image acquisition, and integration into the surgical workflow, based on two illustrative clinical cases. We hypothesized that a miniaturized linear ultrasound probe can be safely introduced into the spinal canal through a standard extended interlaminar approach without enlargement of the bony window and that it enables visualization of relevant intraspinal structures. Technical limitations and potential solutions are also discussed.

## 2. Materials and Methods

### 2.1. Study Design

This study was designed as an observational feasibility study evaluating the intraoperative application of a novel imaging technique.

### 2.2. Ethics Statement

The study describes the intraoperative application of a non-interventional imaging technique during routine clinical care. The ultrasound probe (Fujifilm L51K) was applied in an off-label manner, as it is not specifically approved for intraspinal use. This off-label application was reviewed and approved by the institutional department head and the responsible senior surgeon prior to clinical use. A formal risk assessment was conducted, considering the probe dimensions, material biocompatibility, sterilization compatibility, and the absence of intended direct neural tissue contact. According to institutional guidelines, formal ethics committee approval was not required for this type of observational feasibility report involving an off-label imaging application within the scope of routine surgical care. All patients were informed in detail about the exploratory, non-therapeutic, and off-label nature of the intraoperative ultrasound application and provided written informed consent for both the procedure and the use of anonymized clinical and imaging data for scientific publication.

All procedures were performed in accordance with institutional standards and the principles of the Declaration of Helsinki.

### 2.3. Equipment and Setup

**Figure 1:**
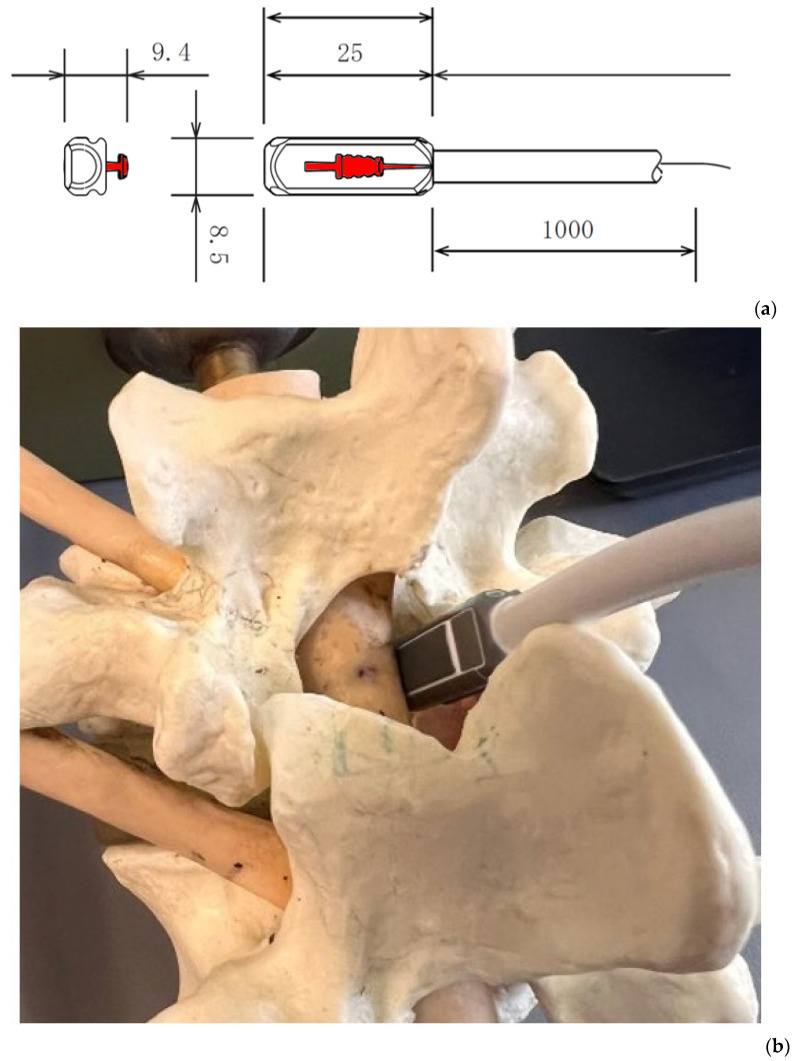

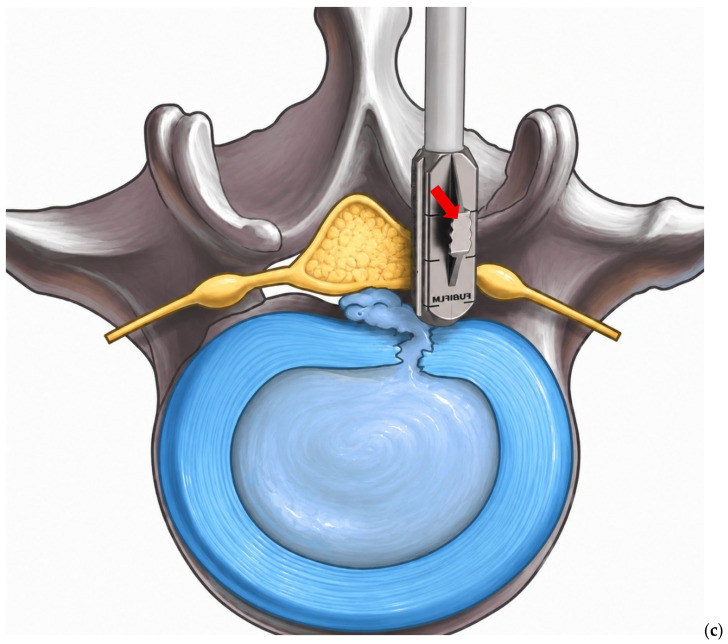
Illustration of the geometry of the Fujifilm L51K probe with dimensions provided in millimeters (**a**), and of its intraspinal positioning as introduced intraoperatively, shown in a representative anatomical model (**b**). The anatomical model demonstrates the probe insertion trajectory through the interlaminar window and its lateral positioning alongside the dural sac, corresponding to the operative orientation used in both clinical cases. Schematic illustration of the intraoperative insertion of the Fujifilm L51K probe (**c**). Note the dorsal fin (highlighted in red in panel (**a**) and indicated by a red arrow in panel (**c**)), which increases the effective probe cross-section and may limit maneuverability within the confined intraspinal space.

Intraoperative ultrasound imaging was performed using a miniaturized linear probe (Fujifilm L51K, Fujifilm Healthcare, Tokyo, Japan; Figure 1) connected to a Fujifilm Arietta A65 ultrasound system. The probe has a transducer head length of approximately 25 mm (Figure 1), providing a 13 mm imaging field and operating in a frequency range of 3–15 MHz. The ultrasound probe (Fujifilm L51K) was applied in an off-label manner, as it is not specifically approved for intraspinal use. According to the manufacturer, the probe is intended for abdominal, small parts, and intraoperative applications; intraspinal use is not among the listed indications. The off-label application was based on a departmental risk assessment considering the probe’s small dimensions, smooth surface geometry, and biocompatible housing materials. The probe geometry and its intraspinal positioning are illustrated in Figure 1.

Standard microsurgical lumbar decompression or microdiscectomy was performed using a minimally invasive interlaminar approach under microscopic visualization.

The probe was sterilized prior to intraoperative use using low-temperature hydrogen peroxide plasma sterilization (H_2_O_2_; STERRAD system). This sterilization method is compatible with the probe materials according to the manufacturer’s reprocessing instructions. The sterilization cycle was verified by chemical indicators. A sterile probe sheath was not used for the following reasons: the probe was fully sterilized using a validated process, no intended direct contact with neural tissue or cerebrospinal fluid occurred, and the use of a sheath would have increased the effective probe diameter, potentially compromising safe insertion through the confined interlaminar corridor. The decision to omit the sheath was made after careful consideration of the risk-benefit balance and is acknowledged as a point requiring further evaluation in future protocols.

Atraumatic probe insertion was ensured through several procedural safeguards. First, dural integrity was confirmed by a transient increase in positive end-expiratory pressure (PEEP), and then the probe was introduced under direct microscopic visualization at all times, allowing the surgeon to monitor the probe tip relative to the dural sac and nerve roots. Second, insertion was performed only after completion of neural decompression and hemostasis, ensuring an unobstructed corridor. Third, the probe was advanced using gentle manual pressure without forced manipulation; any resistance encountered during insertion was considered a stopping criterion, requiring probe withdrawal and repositioning. Fourth, the probe was maintained in a lateral position to minimize mechanical stress on the dural sac. Verification that no dural injury occurred was based on continuous microscopic visualization of the probe tip during insertion and positioning, combined with the absence of visible tissue compression or intraoperative signs of neural or dural irritation, such as muscle twitching.

### 2.4. Probe Insertion and Imaging Protocol

After completion of the primary decompression procedure and achievement of hemostasis, the ultrasound probe was introduced into the spinal canal via the same microsurgical access corridor used for the primary procedure, without further enlargement of the interlaminar fenestration. Probe insertion was performed as the final intraoperative step immediately prior to wound closure. The additional time required for probe insertion, imaging, and withdrawal was estimated at approximately 3–5 min per case. Particular care was taken to avoid forced manipulation or excessive mechanical contact with dural structures. For acoustic coupling, the operative field was filled with sterile 0.9% saline solution prior to ultrasound acquisition. Continuous irrigation with 0.9% saline solution was already routinely used during the bony drilling and fenestration procedures, thereby providing an adequate fluid medium for intraoperative ultrasound transmission within the surgical corridor.

Ultrasound imaging was performed immediately prior to wound closure. The probe was positioned laterally along the dural sac with the transducer face oriented medially toward the thecal sac. Imaging was performed in the transverse (axial) plane along the spinal canal axis, and the probe was slightly rotated to obtain additional axial and oblique views.

Intraoperative ultrasound examinations were performed using acquisition parameters adapted for intraspinal endosonography with the Fujifilm L51K linear probe. Representative imaging settings for the applied examination setup are summarized in Table 1. These settings enabled real-time visualization of the dural sac, intradural nerve roots, and surrounding anatomical structures in both cases. In both cases, the ultrasound probe was inserted atraumatically and guided alongside the dural sac to the level of the posterior longitudinal ligament. From this position, axial imaging planes were obtained and oriented toward the contralateral pedicle and the contralateral lateral recess. The probe could subsequently be slightly angulated and rotated within the spinal canal to achieve multiplanar visualization of the decompression site and adjacent neural structures. Imaging focused on visualization of the dural sac contour, ipsilateral and contralateral nerve roots, the decompression site, and the presence or absence of residual compressive pathology. Adequate decompression was defined qualitatively as: (1) restoration of a smooth, convex dural sac contour without indentation or compression; (2) visualization of freely mobile nerve roots within the thecal sac without displacement or tethering; and (3) absence of residual disc material or bony fragments causing mass effect on neural structures. Image interpretation was performed in real time by the operating surgeon (R.S.), who has prior experience with intraoperative ultrasound in neurosurgical applications.

### 2.5. Outcome Measures

The primary outcome was the technical feasibility of probe insertion and image acquisition through the existing microsurgical corridor. The secondary outcomes included: intraoperative usability (ease of probe handling, angulation, and rotation), integration into the surgical workflow (additional operative time), visualization of neural structures (dural sac, nerve roots, decompression site), and safety (absence of intraoperative and short-term postoperative complications).

## 3. Results

### 3.1. Feasibility and Imaging Outcomes

IOISES was performed in two patients undergoing lumbar spine surgery: one for lumbar disc herniation at L5/S1 and one for multisegmental lumbar spinal canal stenosis with maximum at L3/4.

**Figure 2:**
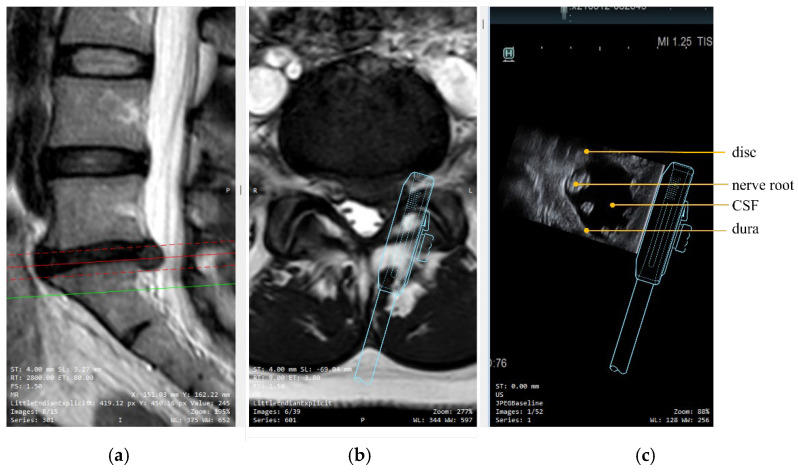
MRI showing lumbar predominantly left-sided median disc herniation at L5/S1, median-to-left-sided emphasis: (**a**) sagittal view, (**b**) axial view, (**c**) intraoperative intraspinal endosonographic finding after sequestrectomy demonstrating complete dural decompression. The ultrasound image (**c**) was obtained with the probe positioned laterally in the spinal canal, with the transducer face oriented medially toward the thecal sac. The ultrasound probe (blue) was schematically superimposed onto the axial MRI and ultrasound image. IOISES enabled visualization of the dural sac as a hyperechoic linear structure, the ipsilateral and contralateral nerve roots as hypoechoic rounded structures, and the surrounding cerebrospinal fluid (CSF) as an anechoic space.

**Figure 3:**
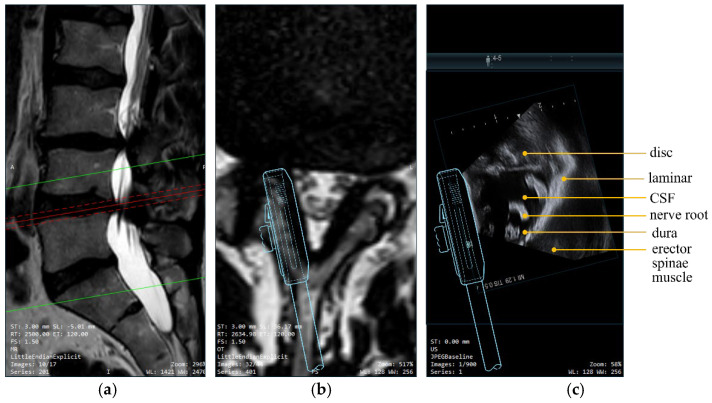
MRI showing bisegmental spinal canal stenosis at L3/4 and L4/5: (**a**) sagittal view, (**b**) axial view, (**c**) intraoperative intraspinal endosonographic finding in segment L4/L5 after decompression demonstrating complete dural decompression, ultrasound probe visible from the left in the ultrasound image. The ultrasound image (**c**) was acquired after microsurgical decompression. The ultrasound probe (blue) was schematically superimposed onto the axial MRI and ultrasound image. IOISES enabled visualization of the disc, the dural sac as a hyperechoic linear structure, the ipsilateral and contralateral nerve roots as hypoechoic rounded structures, the surrounding cerebrospinal fluid (CSF) as an anechoic space, and the lamina as a hyperechoic surface with posterior acoustic shadowing.

In both cases (100%), the ultrasound probe was successfully introduced into the spinal canal through the existing microsurgical corridor without requiring extension of bony decompression. IOISES enabled clear visualization of the dural sac as a hyperechoic linear structure, the ipsilateral and contralateral nerve roots as hypoechoic rounded structures, the surrounding cerebrospinal fluid (CSF) as an anechoic space, the lamina as a hyperechoic surface with posterior acoustic shadowing, and the decompression site itself (Figure 2 and Figure 3). No residual compression was identified intraoperatively.

The red solid line in the sagittal image indicates the level of the corresponding axial slice. 

### 3.2. Intraoperative Usability

Probe handling, angulation, and rotation within the spinal canal were feasible without forced manipulation. The ultrasound probe could be rotated by approximately 20° in both directions within the spinal canal, allowing adequate visualization without causing tension or obstruction. No intraoperative complications attributable to probe use were observed, including no cerebrospinal fluid leak, no neurological worsening, no visible tissue damage, and no muscle twitching. The technique was integrated into the surgical workflow with an estimated additional operative time of 3–5 min per case. During short-term postoperative follow-up, no procedure-related complications occurred in either patient, including no wound infection, no new neurological deficit, and no recurrent symptoms attributable to the ultrasound application.

### 3.3. Technical Considerations and Instrument Design

The L51K probe includes a dorsal fin designed to facilitate manipulation with a grasper during abdominal and robotic applications. In the intraspinal environment, this fin is not functionally required and may limit maneuverability due to spatial constraints within the spinal canal. A finless variant of the probe would provide an even slimmer profile and may improve handling and safety by reducing mechanical interference with surrounding anatomical structures (Figure 1).

## 4. Discussion

Ongoing advances in microfabricated ultrasound transducers have enabled substantial miniaturization of ultrasound probes. Highly miniaturized ultrasound transducers have already been demonstrated in several catheter- and endoscope-based imaging systems. Intravascular ultrasound (IVUS) incorporates compact transducer arrays into millimeter-scale catheters, while endobronchial ultrasound (EBUS) integrates ultrasound probes into bronchoscopic devices to allow transmural imaging of mediastinal structures [4,5]. In addition, recent work has demonstrated the integration of miniaturized ultrasound transducer arrays directly into neurosurgical needles, enabling real-time imaging within instruments with diameters of only a few millimeters [6].

These developments underscore the translational potential of further miniaturization of ultrasound probes and support the feasibility of intraoperative ultrasound applications in confined surgical corridors.

Intraoperative spinal ultrasound itself has been described for several decades [1,2]. However, most previously reported applications rely on an extraspinal approach requiring sufficiently wide laminectomy windows to achieve adequate acoustic coupling. This anatomical constraint has limited its integration into modern minimally invasive spinal surgery. The broader role of intraoperative ultrasound in spinal surgery—including visualization of intradural and intramedullary lesions requiring more extensive laminectomy or laminotomy—has been comprehensively reviewed by Vasudeva et al. [7], while its use in surgical navigation has been summarized in a systematic review by Patel et al. [8].

The present study introduces IOISES as a modification of this concept. By enabling direct probe placement within the spinal canal through a microsurgical corridor, this approach represents a conceptual shift from conventional extraspinal ultrasound toward an intraspinal imaging paradigm. The key technical difference compared to prior intraoperative spinal ultrasound is not merely miniaturization, but also the ability to position the transducer inside the spinal canal, thereby circumventing the need for a wide bony window for acoustic coupling. Conventional extraspinal ultrasound requires a laminectomy window large enough to accommodate the probe footprint and provide an acoustic path through the posterior elements, whereas IOISES uses the existing interlaminar corridor. Visualization of the dural sac and traversing nerve roots can thus be achieved without extending the bony decompression. This approach may be particularly relevant in minimally invasive procedures, where visual access is intentionally restricted and intraoperative confirmation of complete decompression can be challenging.

Incomplete decompression and residual disc material have been implicated as potential contributors to persistent or recurrent symptoms following lumbar surgery. Reported revision rates after lumbar disc surgery range between approximately 5% and 25%, with residual disc material representing a relevant contributing factor [3,9]. It is conceivable that intraoperative intraspinal imaging could assist in detecting residual compressive pathology during the primary procedure; however, the present study was not designed to evaluate this, and no conclusions regarding the effect of IOISES on revision rates or clinical outcomes can be drawn from these two cases.

The present report is limited to two illustrative cases and does not permit conclusions regarding clinical outcomes or diagnostic accuracy. Nevertheless, the qualitative concordance between the intraoperative ultrasound findings and preoperative MRI in both cases supports the technical validity of the imaging approach as a proof of concept. Future studies with systematic correlation between the IOISES findings and postoperative imaging (e.g., MRI) will be necessary to validate the diagnostic accuracy of this technique.

Hypothetically, the technique could also be explored for other intraspinal pathologies such as epidural abscesses, juxtafacet cysts, or intraspinal tumors, although these represent speculative indications that would require dedicated feasibility testing. Similarly, intraoperative intraspinal ultrasound may hypothetically contribute to radiation-free spinal navigation by providing real-time anatomical imaging that could be fused with preoperative MRI datasets; however, this remains a theoretical application at present.

### 4.1. Limitations and Technical Constraints

Several limitations must be acknowledged. First, the current report reflects initial technical feasibility in only two patients and represents an illustrative proof-of-concept description rather than a systematic clinical series. Reproducibility across different surgeons, anatomical variations, and spinal levels remains to be established through larger studies with standardized protocols.

Second, device-related safety considerations are critical. The Fujifilm L51K probe is used off-label for an intraspinal application, as it is not specifically approved for this indication. The device is not intended for direct contact with neural tissue or cerebrospinal fluid according to the manufacturer’s specifications. Accordingly, IOISES must not be performed in cases of dural injury where CSF exposure would violate device approval conditions. A comprehensive safety protocol including defined stopping criteria, required microscopic visualization, and avoidance of forced manipulation was followed in both cases (see Section 2). Nonetheless, the safety profile of repeated intraspinal probe insertion requires further systematic evaluation.

In addition, several methodological and practical limitations should be considered. The use of IOISES may be associated with a learning curve, particularly with respect to probe handling within the confined intraspinal space, optimization of image acquisition, and real-time interpretation of sonographic findings. In the present cases, the operating surgeon (R.S.) had prior experience with intraoperative ultrasound in neurosurgical applications; however, the transferability of this technique to surgeons without prior ultrasound training has not been assessed.

Third, interpretation of intraoperative ultrasound images is operator-dependent and requires familiarity with spinal sonoanatomy. In the present cases, image interpretation was performed in real time by the operating surgeon. Variability in image interpretation may affect reproducibility and limit generalizability across different surgical settings. Whether the same images could be consistently obtained across cases, across spinal levels, or across different operators has not been assessed and represents an important area for future standardized reproducibility studies.

Furthermore, the present report is based on a qualitative assessment of feasibility and intraoperative utility, without quantitative metrics. Objective parameters for decompression adequacy, image quality, or diagnostic accuracy were not defined prospectively and should be addressed in future studies. Importantly, no postoperative imaging (e.g., MRI) was performed to validate the intraoperative sonographic impression of adequate decompression. The absence of postoperative imaging correlation is a significant limitation, and future studies should include systematic postoperative validation to determine the diagnostic reliability of IOISES findings.

Additional technical limitations include the constrained field of view inherent to the small transducer footprint (13 mm imaging field), which limits the area of visualization per scan position and requires sequential imaging with probe repositioning. Image artifacts related to the confined surgical corridor, including reverberation and near-field artifacts, may further limit image quality. The limited penetration depth of a high-frequency small linear probe may also restrict visualization of deeper anatomical structures. Finally, the dorsal fin of the current probe model, designed for abdominal and robotic applications, may limit maneuverability within the confined intraspinal space. A finless or spine-specific variant could improve handling characteristics and further enhance procedural safety.

### 4.2. Future Directions

Future investigations should focus on standardized handling and imaging protocols, development of an anatomical ultrasound atlas correlated with MRI, and prospective evaluation of usability, reproducibility, and inter-operator reliability. Systematic correlation of intraoperative IOISES findings with postoperative MRI and clinical outcomes will be essential to establish the diagnostic accuracy and clinical value of this technique. Larger prospective clinical studies will be required to determine whether IOISES can influence intraoperative decision-making or contribute to improved surgical outcomes. Development of a dedicated spine-specific ultrasound probe with optimized dimensions, an atraumatic tip design, and removal of the dorsal fin would further facilitate clinical translation.

## 5. Conclusions

This pilot study demonstrates that IOISES using a miniaturized linear ultrasound probe introduced through a microsurgical lumbar corridor is technically feasible and enables real-time intraspinal visualization without extension of the bony decompression. The strongest contribution of this work is the demonstration that probe insertion and image acquisition are achievable through a standard microsurgical interlaminar approach. Probe design optimization, standardized imaging protocols, and systematic clinical evaluation in larger prospective cohorts are warranted before the clinical role of IOISES in modern spinal surgery can be determined.

## Figures and Tables

**Table 1:** Representative Ultrasound Acquisition Parameters for Intraoperative Intraspinal Endosonography Using the FUJIFILM L51K Linear Probe with the Arietta 65 Ultrasound System.

Parameter	Setting Used for IOISES-L51K	Scientific Description
Ultrasound preset/examination mode	IOISES-L51K	Dedicated preset optimized for intraoperative intraspinal endosonography using the Fujifilm L51K linear probe.
Frequency setting	15 MHz Rx HdT harmonic mode	High-frequency reception setting with harmonic tissue imaging enabled to improve spatial resolution and tissue contrast.
Imaging depth	25 mm	Shallow imaging depth optimized for visualization of intraspinal anatomical structures within the surgical corridor.
Gain	BG 56–61	Image amplification settings adjusted individually for optimal visualization and contrast during image acquisition.
Doppler mode	Off	Pure B-mode imaging without Doppler application.
Harmonic imaging	Enabled (HdT)	Harmonic tissue imaging activated to reduce artifacts and improve image clarity.
Focal zone	Single focal zone at approximately 12 mm depth	Focal region positioned at the level of the target intraspinal structures to maximize lateral resolution.
Dynamic range	BD 76	Dynamic range setting used to balance tissue contrast and grayscale differentiation.
Probe orientation	Left-sided orientation	Probe marker oriented to the left side during acquisition.
Irrigation/acoustic coupling medium	0.9% saline solution	Sterile saline/water used as acoustic coupling medium to facilitate ultrasound transmission within the surgical field.

## Data Availability

The original contributions presented in this study are included in the article. Further inquiries can be directed to the corresponding author.

## Abbreviations

The following abbreviations are used in this manuscript:

IOISES	Intraoperative Intraspinal Endosonography
MHz	Megahertz
MRI	Magnetic Resonance Imaging
